# Early and midterm results of transapical and right axillary artery cannulation for acute aortic dissection

**DOI:** 10.1186/s13019-014-0202-9

**Published:** 2015-01-09

**Authors:** Takamitsu Terasaki, Tamaki Takano, Taishi Fujii, Tatsuichiro Seto, Yuko Wada, Yoshinori Ohtsu, Kazunori Komatsu

**Affiliations:** Department of Cardiovascular Surgery, Shinshu University School of Medicine, 3-1-1 Asahi, Matsumoto, 390-8621 Japan

**Keywords:** Aortic dissection, Cannulation, Aortic surgery, Complication

## Abstract

**Background:**

We combined transapical cannulation and right axillary artery cannulation in the repair of acute type A aortic dissection in order to reduce mortality and morbidity in the presence of risk of malperfusion. Early and midterm outcomes were evaluated.

**Methods:**

Between October 2009 and March 2012, 23 aortic dissection patients (age, 54.3 ± 13.5 years) received graft replacement using a combination of transapical and right axillary artery cannulation. Preoperative malperfusion was present in 16 patients (69.6%). Cardiopulmonary bypass was initiated with axillary artery cannulation applied via the right axilla and right atrial drainage, then aotric cannulation applied via the left ventricular apex. We retrospectively investigated mortality and morbidity as well as cardiac function, which were evaluated echocardiographically during hospitalization and once a year postoperatively.

**Results:**

All patients received total arch replacement. In-hospital mortality was 4.3%, and no patient developed intraoperative malperfusion. Intraoperative stroke occurred in one patient (4.3%), and three patients (13.0%) suffered from delayed stroke (10–24 days). These delayed strokes might have resulted from cardiogenic thrombus, although no intracardiac thrombus was found. Mean ejection fraction was 66.1 ± 10.9% in the early postoperative period and 73.1 ± 8.7% midterm. There was no left ventricular asynergy or intracardiac thrombus seen on either early or midterm echocardiography.

**Conclusion:**

Transapical cannulation with right axillary artery cannulation is a safe and effective procedure that can reduce operative risk associated with aortic dissection. Although transapical cannulation does not appear to impair cardiac function, it may confer a risk of delayed stroke.

## Background

It is sometimes difficult to choose a cannulation site that will avoid organ malperfusion in acute type A aortic dissection (AAD) when the true lumen is compressed by a false lumen and femoral artery cannulation presents risks of organ malperfusion and brain infarction because of retrograde blood flow and embolism [1,2].

Transapical cannulation (TAC) had been introduced as a useful application of cardiopulmonary bypass (CPB) in the presence of a severely atherosclerotic ascending aorta [3]. Some authors have reported that TAC can be an alternative technique for cannulation during AAD repair, especially since the introduction of transesophageal echocardiography (TEE) to ensure the location of the cannula [4-7], but mortality (7.7–18.8%) and rate of cerebral complications (5.8–15.8%) remain high. The axillary artery is an alternative cannulation site that can provide antegrade blood flow during CPB [8], and its efficacy at reducing the risk of stroke during AAD repair has been documented [9,10].

We combined TAC and right axillary artery cannulation (RAAC) for the repair of AAD in the presence of a risk of malperfusion. In this study, we evaluated early and midterm outcomes of AAD repair employing a combination of TAC and RAAC.

## Methods

Between October 2009 and March 2012, 71 AAD patients underwent aortic graft replacement in Shinshu University Hospital. Of 71 patients, 24 (33.8%) received graft replacement with combined TAC/RAAC. In one patient, TAC was abandoned intraoperatively because of left ventricle distention from massive aortic valve regurgitation, so 23 of 24 TACs (95.8%) were completed and analyzed.

Indications for TAC and RAAC were one or more contraindications for femoral cannulation, including iliofemoral artery dissection and presence of abdominal aortic or iliac artery aneurysm. Mean patient age was 54.3 ± 13.5 years, and male/female ratio was 15/8. Clinical background and preoperative conditions are shown in Table 1. Preoperative malperfusion of one or more organs was present in 16 patients (69.6%).

**Table 1:** **Preoperative patients characteristics**

	**Cases (n = 23)**
**Age (y)**	**54.3 ± 13.5**
**Male/Female sex**	**15/8**
**Loss of conciousness**	**5 (20.8%)**
**Aortic regurgitation > mild**	**6 (25.0 %)**
**Pericardial efusion**	**5 (21.7%)**
**Malperfusion**	**16 (69.6%)**
**Cerebral**	**8 (34.8%)**
**Myocardial**	**2 (8.7%)**
**Limb**	**10 (43.5%)**
**Mesenteric**	**1 (4.3%)**

TAC: trans apical cannulation, RAAC: right axillary artery cannulation.

We performed total arch replacement in patients aged ≤ 70 years. Hemi-arch replacement was performed in patients aged over 70 when the initial tear was found at the ascending aorta. RAAC was performed by exposing the right axillary artery through a 4- to 6-cm skin incision in the right armpit and inserting a 10- to14-Fr DLP® one-piece pediatric arterial cannula (Medtronic, Minneapolis, MN) through the artery. Right atrial drainage achieved using a single venous cannula, and the patient was then placed on CPB. TAC was accomplished with a 21-Fr Soft-Flow® Extended arterial cannula (Terumo, Tokyo, Japan) through the left ventricular apex. After the location of cannula tips was confirmed by TEE, blood flow was increased to maintain adequate perfusion.

To protect the brain and abdominal organs, we used selective cerebral perfusion and hypothermic circulatory arrest with a rectal temperature of 25°C. Open distal anastomosis was performed first, and total CPB was then restarted through a side branch of the vascular graft. After the proximal anastomosis, the apical incision was closed using 4–0 polyvinylidene fluoride suture with felt pledgets. Neck vessels were individually reconstructed during rewarming.

We retrospectively investigated in-hospital mortality and morbidity. Cardiac function and thrombus around the left ventricular apex was evaluated using echocardiography postoperatively during the hospital stay and once a year after discharge from the hospital. Statistical comparison of postoperative left ventricular ejection fraction between early and midterm periods was conducted using the Student *t* test using IBM SPSS statistics 21.0 software (IBM Corp, Armonk, NY USA). The Shinshu University School of Medicine Institutional Ethics Committee approved this study.

## Results and discussion

Total arch replacement was performed in all patients. Concomitant procedures were root reconstruction in four patients (17.4%), aortic valve replacement in one (4.3%), and coronary artery bypass in three (13.0%) (Table 2).

**Table 2:** **Operation and results**

		**Cases (n = 23)**
**• Procedures**	**Total arch replacement**	**23 (100%)**
**• Concomitant procedures**	**Root recontraction**	**4 (17.4 %)**
	**Aortic valve replacement**	**1 (4.3%)**
	**CABG**	**3 (13.0%)**
**• Clinical results**	**In-hospital mortality**	**1 (4.3%)**
	**Intraoperative srtoke**	**1 (4.3%)**
	**Delayed stroke**	**3 (13.0%)**
	**Intraoperative organ malperfusion**	**0**
	**Newly required hemodialysis**	**0**
	**Revision for bleeding**	**2 (8.7%)**
	**Prolonged ventilation ( >72 h)**	**3 (13.0%)**
	**Hospital stay (d)**	**35.7 ± 18.6**

CABG: Coronary artery bypass grafting.

In-hospital mortality was 4.3%; one patient died on the 6th postoperative day because of low-output syndrome. He had presented with severe bilateral limb ischemia and undergone total arch replacement with coronary artery bypass grafting (Table 2).

No patient developed intraoperative malperfusion of the limbs or abdominal organs requiring additional operation. One patient, whose celiac artery was obstructed by the dissection on preoperative computed tomography, had temporary liver dysfunction postoperatively. Intraoperative stroke occurred in one patient (4.3%), and three patients (13.0%) suffered from stroke on postoperative days 10–24 (Table 3), although they recovered to care for themselves and discharged from hospital by walk.

**Table 3:** **Patient characteristics of delayed stroke cases**

**Patient**	**Age (y)**	**Sex**	**Operation**	**Onset of stroke (d)**	**Atrial fibrillation**	**Intracardiac thrombus**	**Cervical artery dissection**
**1**	**35**	**Male**	**Total arch**	**24**	**no**	**no**	**no**
**2**	**63**	**Male**	**Total arch**	**18**	**no**	**no**	**no**
**3**	**66**	**Female**	**Total arch**	**10**	**no**	**no**	**yes**

Postoperative echocardiography was performed in 21 patients (95.5%) 12.4 ± 3.8 days after the operation. Mean ejection fraction was 66.1 ± 10.9%. No new left ventricular asynergy or intracardiac thrombus developed postoperatively (Table 4).

**Table 4:** **Echocardiography data**

**Variables**	**Early period (n = 21)**	**Midterm period (n = 17)**	**P value**
**LVEF (%)**	**66.1 ± 10.9**	**73.1 ± 8.7**	**0.053**
**LVDd (mm)**	**44.6 ± 7.0**	**46.7 ± 4.3**	**0.44**
**Aortic regurgitation > mild (n)**	**0**	**0**	
**Asynergy (n)**	**1**	**1**	
**Pseudoaneurysm (n)**	**0**	**0**	
**Intracardiac thrombus (n)**	**0**	**0**	

LVEF: Left ventricular ejection fraction, LVDd: Left ventricular diastolic dimension.

No patient died or developed stroke during the follow up period (23.6 ± 8.5 months). Two patients received additional vascular surgery (one for aneurysm of the thoracic descending aorta and the other for abdominal aortic aneurysm). Transthoracic echocardiography was able to be performed in 17 of 22 patients (77.3%) during the midterm period, 15.6 ± 8.1 months after the operation. Mean ejection fraction was 73.1 ± 8.7%. We found no left ventricular asynergy, intracardiac thrombus, or left ventricular aneurysm (Table 4).

Surgical mortality for AAD has been reported to be 23.8% [11], increasing to 43.7% when the patient presents with organ malperfusion preoperatively [12]. Mortality reaches 63.2% in cases complicated by mesenteric malperfusion [13]. Organ ischemia, caused by occlusion by a dissection flap or thrombus in the branch arteries, affects 15–40% of patients with AAD [14]. In the present study, in-hospital mortality was 4.3% and no patient developed intraoperative malperfusion, even though the rate of preoperative malperfusion was 69.6% and relatively higher than that seen in the reports mentioned above. The combination of RAAC with TAC seemed to prevent intraoperative malperfusion during AAD surgery. RAAC is effective to reduce the risk of stroke, but it could not achieve total blood flow alone. The initial perfusion with RAAC would make true lumen more patent and help establish total circulatory support after TAC is added. In a report on TAC perfusion without RAAC for AAD repair, Matsushita et al. described the occurrence of malperfusion in four out of 52 patients on initiation of CPB [7]. In that study, applying TAC may have caused hemodynamic instability owing to hypotension and arrhythmia occurring during cannulation and TEE confirmation of positioning. Although TAC is not a technically demanding procedure, the left ventricular apex does need to be lifted up. However, no patients presented any injury in the fragile aorta of AAD in applying TAC. We were able to establish CPB under safe and stable conditions with the help of RAAC prior to TAC. RAAC can provide appropriate support for the application of TAC, especially for hemodynamically unstable patients.

The stroke rate in the present study is comparable to the 5.8–15.8% observed in previous studies using TAC alone for AAD surgery [5,7], with one case of intraoperative stroke (4.3%) and three cases of stroke occurring more than 10 days postoperatively (13.0%). Another study reported that delayed stroke occurred in 25.3% of patients with cervical artery dissection without TAC [15]. In the present study, one of three patients with delayed stroke presented with cervical artery dissection; the other two cases did not. These delayed strokes may have been be caused by cardiogenic thrombus, although no thrombus was found in cardiac chambers on postoperative transthoracic echocardiograpy (TTE). It is important to be aware of the risk of delayed stroke after AAD surgery with TAC.

We found no LV asynergy or pseudoaneurysm on the apex and no aortic valve regurgitation in the early and midterm postoperative periods by TTE. Neither pseudoaneurysm nor intracardiac thrombus was seen on follow up echocardiography. Left ventricular ejection fraction increased slightly in the midterm period even though the left ventricular apex had received iatrogenic injury from passing of the cannula and suturing. There are few reports of postoperative cardiac function after surgery employing TAC. In our experience, TAC does not affect cardiac function in the early and midterm periods after repair of AAD, but a long-term follow-up study is warranted.

## Conclusions

In conclusion, the combination of TAC and RAAC is safe and effective at reducing the risk of malperfusion in AAD. TAC may not affect cardiac function in the early and midterm postoperative periods but may confer a stroke risk after 10 days postoperatively.

## Abbreviations

AAD: Acute type A aortic dissection
TAC: Transapical cannulation
CPB: Cardiopulmonary bypass
RAAC: Right axillary artery cannulation
TTE: Transthoracic echocardiograpy
